# High-Res Acoustic and Environmental Data to Monitor *Bombus dahlbomii* Amid Invasive Species, Habitat Loss

**DOI:** 10.1038/s41597-025-04877-1

**Published:** 2025-04-01

**Authors:** Patrick Chwalek, Marie Kuronaga, Isamar Zhu, Sophia Montague, Victoria Campopiano Robinson, Josefina Lohrmann, Cristian Alfonso Villagra Gil, David Susič, Anton Gradišek, Johannes Schul, Joseph A. Paradiso, Marina Arbetman

**Affiliations:** 1 Responsive Environments, MIT Media Lab, Cambridge, MA USA; 2 Instituto de Investigaciones en Biodiversidad y Medioambiente (INIBIOMA), Universidad Nacional del Comahue y CONICET, Bariloche, Argentina; 3 Instituto de Entomología, Universidad Metropolitana de Ciencas de la Educación, Santiago, Chile; 4 Jožef Stefan Institute, Jamova cesta 39, SI-1000 Ljubljana, Slovenia; 5 Division of Biological Sciences, University of Missouri, Columbia, MO USA

**Keywords:** Biodiversity, Invasive species, Electrical and electronic engineering, Population dynamics

## Abstract

The decline of the endemic Patagonian bumblebee (*Bombus dahlbomii*) as a result of invasive species and habitat loss, among other stressors, has raised significant conservation concerns for the species and the ecosystem it inhabits. In order to monitor this endangered species, traditional methods are limited by labor-intensive visual surveys or lethal sampling methods. We applied passive acoustic monitoring (PAM) as a non-invasive alternative to conventional monitoring techniques to collect a comprehensive dataset of the soundscape of Puerto Blest, Argentina, focusing on bumblebee bioacoustics and environmental variables. Our dataset, collected using custom stereo acoustic recorders, includes audio, temperature, humidity, and gas concentration data from twelve locations over six days, covering different weather conditions. Annotations marking native and invasive bee segments provide insights into the ecology of *B. dahlbomii* and its interactions with invasive species, *Bombus terrestris*. This dataset facilitates the development of machine learning models for monitoring *Bombus* populations, crucial for conservation efforts. Additionally, our robust data annotation techniques enhance the dataset’s reliability for future modeling work.

## Background & Summary

Globally, bumble bees are threatened by a range of environmental stressors including habitat degradation, climate change, pesticides, invasion of nonnative species, and pathogen transmission^1–3^. Surveys from Europe, North America, and South America indicate a temporal shift in the relative abundance and spacial shift in the distribution of *Bombus* species^2,4^. Biological invasions of managed *Apidae* acquired for agricultural pollination, such as bumblebees, was named in the top 15 emerging issues for global conservation and biological diversity in 2017^5^. In Patagonia, spanning southern regions of Chile and Argentina, the large garden bumblebee (*Bombus ruderatus*) and the buff-tailed bumblebee (*Bombus terrestris*) have extended their range into the habitat of the native Patagonian bumblebee (*Bombus dahlbomii*) since their initial introduction through agriculture and the international bee trade, leaving *B. dahlbomii* classified as endangered by the IUCN^6^. Populations of *B. dahlbomii* have faced a historical decline that is projected to continue in areas beyond where alien bumblebees have been introduced^7,8^. The commercial rearing of *B. terrestris* imported to Chile for pollination has led to an invasion which results in the foraging superposition between native and exotic bumblebees and pathogen spillover from these bees to wild populations of *B. dahlbomii*, posing significant conservation risks to this emblematic species^9–11^.

Puerto Blest, Argentina, is home to *B. dahlbomii*, recognized as one of the largest bumblebee species, and colloquially known as the “moscardón” in Chile and “mangangá” in Argentina. These bees are highly ecologically significant, playing a crucial role in the pollination of a diverse range of flowering plants, including economically important crops and endangered species^12–14^. Patagonia’s native plants are impacted by *B. dahlbomii*’s decreased visitation, apparent in reduced metrics of reproduction like fruit set^15^. Endemic bumblebees are more efficient pollinators to many native plant species than invaders, with larger body sizes, increased frequency and duration of visits to flowers, and a higher quantity and quality of pollen^16–18^. Their decline raises concerns not only for the conservation of this iconic species but also for the stability of Patagonia’s interconnected ecosystems and the services they provide^19^.

On a smaller scale, invasive *B. terrestris* are not only directly competing with endemic *B. dahlbomii*, but also altering the landscape in which they forage. *B. terrestris* have constructed an ecological niche in their new ecosystem by changing their feeding behaviors to bite holes at the base of *Fuchsia magellanica* flowers rather than typical front visits. This behavior favors the survival of invasive bee species, as nectar robbing (via biting holes) increases foraging returns, depletes resources for other species, and provides continued access to nectar until the flower wilts^20^. Nectar robbing by invasive bees can damage flowers and disrupt plant-pollinator mutualisms by bypassing a flower’s reproductive structures, raising concern for plant reproduction and population viability^15,21^. *B.dahlbomii* are responding to *B. terrestris*’ ecological niche construction by secondary nectar robbing, buffering *B. terrestris*’ competitive advantage, which could promote species coexistence. Still, this niche and potential distribution overlap leads species distribution models to forecast an ongoing population decrease of *B. dahlbomii* in the near future^8^. These complex and evolving ecological interactions necessitate ongoing monitoring in the field to better track *Bombus* spp. populations and biodiversity metrics.

Long-term and large-scale population assessments are essential for understanding shifts in *Bombus* species community compositions and their ecological consequences^22^. Conventional techniques for monitoring *Bombus* species, such as netting and trapping, can interfere with natural behaviors and frequently result in the death of the bees being studied. Conversely, non-lethal transect walks, the most common monitoring method, are laborious and involve trained individuals identifying bees along set paths, a practice limited by visibility and the observer’s ability to discern species, especially in dense vegetation or low light conditions^23,24^. Some bee species are indiscernible by sight in the field, so they are grouped together while monitoring via transect walks^23^. Netting and trapping, while effective for detailed species composition and abundance data, require significant effort and expertise^25^. Pan traps, which use colored bowls filled with soapy water to passively collect bees, offer a low-effort method but result in bee mortality, which is undesirable for conservation-focused studies^26^. Additionally, pan traps provide an accumulation of insects over a given period but do not offer regular and systematic sampling of the chosen site. While species distribution models provide valuable insights for population scale estimations, they are not sufficient for concrete and comprehensive bee monitoring. Given these limitations, passive acoustic monitoring (PAM), ecological monitoring through automated sound recording, presents a potential alternative, when coupled with machine learning, to obtain accurate and applicable data on bee populations and their interactions^27–29^. PAM allows for non-invasive, continuous data collection on bee diversity and buzz abundance across various environments with limited species diversity, enhancing the scope and energetic payoff of ecological studies and conservation efforts^30^.

The provided acoustic data and local temperature, humidity, and gases (i.e., volatile organic compounds) can support ecophysiology and behavioral research on how different bee species may display different thermal or temporal activity peaks or behaviorally adapt to abiotic conditions, which in turn affects functional diversity and species richness. In Puerto Blest, we observed less bumblebee activity on colder, wetter days, suggesting that temperature and humidity influence bee behavior in the area. Elevated temperatures can reduce the number of bumblebee foraging trips and time spent foraging, which is increasingly relevant with ongoing global warming^31,32^. Environmental humidity interacts with bumblebee ability to detect floral humidity, as floral humidity signals can masked by environmental humidity at higher ranges, as well as whether bumblebees collect pollen or nectar.^33,34^. Air pressure also impacts bee activity levels and foraging efficiency^35^. Volatile organic compounds are produced by flowering plants to attract pollinators, while carbon dioxide (CO_2_) and ozone (O_3_) alter these volatile organic compound profiles, impacting pollinator interactions^36^. Additionally, environmental data is valuable for building multimodal machine learning models for buzz classification, trained by both acoustic and environmental data in tandem.

Bioacoustic data and machine learning have already been used as a non-invasive and efficient way to identify bumblebees and track their behavior^37–39^. Unique acoustic signatures from flight buzzing sounds can be leveraged to monitor bees with machine learning algorithms. Gradišek *et al*. have identified queens and worker bees from different bee species, as well as behaviors of the worker *Bombus hypnorum* using random forest algorithms trained on bioacoustic data^38^.

Similar species detection technology includes BirdNET and ANIMAL-SPOT, machine learning models for the detection and classification of animal species. BirdNET is targeted specifically towards avian sounds collected through citizen science that claims to be able to identify over 6,000 bird species^40,41^. Using crowdsourced data, BirdNet is repeatedly improved, and could be retrained with new data to be applied to different taxa. ANIMAL-SPOT, a machine learning framework for biologists to adapt, targets more generalized acoustic identification to be extended to many kinds of vocalizing animals. This technology aims to provide the basis to detect and identify animals based on taxonomic group, species, or call type^42^. With these models adapted and applied to this dataset as a mode of species detection, we can employ machine learning to closely monitor *Bombus* spp. while minimizing the effort required to monitor them. By training machine learning models on acoustic and environmental data from Puerto Blest, we can develop robust tools for non-invasive ecological monitoring. This approach promotes continuous and scalable monitoring capabilities, crucial for conservation efforts and understanding complex ecological dynamics.

In this paper, we present a unique dataset of the soundscape in Puerto Blest using custom-made, low noise, stereo acoustic recorders, with specific emphasis on bumblebee bioacoustics. This dataset includes audio, temperature, humidity, and gas concentration data, collected simultaneously. The audio data includes continuous audio from 12 locations over 6 days within the same area, as well as annotated audio segments marked Native/Invasive with respect to identified bees. We also present the use of semi-automatic methods as a new technique to annotate and standardize PAM data collection. This data is applicable for developing methods for insect detection and classification in addition to its use for bumblebee monitoring in Patagonia. We discuss the usefulness of this dataset for scientific research, namely monitoring large temporal series of a low abundant species, as well as to document the bioacoustics of an endangered species in high resolution.

## Methods

### Data Collection

The data collection took place in Puerto Blest, Argentina, between March 10-15, 2024. This work was done under the National Park Administration (APN) permit #1839; IF-2024-14494813-APN-DRPN#APNAC. We collected data over various locations across Puerto Blest using custom recorders equipped with acoustic and environmental sensors that were constructed at the MIT Media Lab. The use of custom recorders allows the team to easily synchronize all data streams and field annotations, as well as simplify the deployment process by reducing the quantity of equipment. Each recorder features two electret microphones (AOM-5024L-HD-R) spaced 144 millimeters apart, encapsulated in a cylinder covered with SAATI fabric (B260HY) to dampen wind noise without significantly diminishing the quality of the recordings. Each microphone signal was sampled at 48 kHz and put through a 20 dB preamplifier, then synchronously sampled with a 24-bit analog-to-digital converter. The signal was digitally amplified by 15 dB before recording the 16 most significant bits (MSB) in a .WAV file. Each device also features an integrated environmental sensor (BME688) which uses Bosch’s Sensortec Environmental Cluster (BSEC) API configured using Bosch’s 28-day, 3.3V, 300s sample rate sensor calibration file. The BME688 sensor outputs a variety of sensing dimensions, but for the purpose of this study we configured it to output raw temperature, pressure, humidity, and equivalent carbon dioxide (CO_2_) (Table 1). All acoustic recordings have accompanying environmental data. We also provide supporting data from a nearby weather station that reports on an hourly basis.

**Table 1:** Sensor Sample Rates and Resolutions.

Sensor	Sample Rate (Hz)	Resolution (Accuracy)
Acoustic (2-channel)	48,000	16-bit ^1^
Temperature	0.2	0.01°C (+/ − 0. 5°C)
Relative Humidity	0.2	0.008% (+/ − 3%)
Pressure	0.2	0.18 Pa (+/ − 0.12 hPa)
Gas	0.2	reference BME688 datasheet

Sound pressure level calibration was not performed but sensor was tuned for the Puerto Blest area to not saturate the acoustic signal when recording natural sounds.

Each device is equipped with a Bluetooth radio that allowed the field team to connect to each one using a custom iOS application to quickly annotate any invasive or native bee sightings. During an annotation session when bees were most active, a team member positioned themselves near the target device and pressed a button on the iOS application that corresponded to the species of bee that was observed (i.e., *B. dahlbomii* or *B. terrestris*). This action was transmitted to the connected recorder which recorded a matching timestamp, allowing us to easily correlate to the timestamp of the recorded audio and extract a 10 second excerpt. This method is a significant improvement from traditional asynchronous manual methods, where paper observations are correlated to recorded acoustic data.

**Fig. 1:**
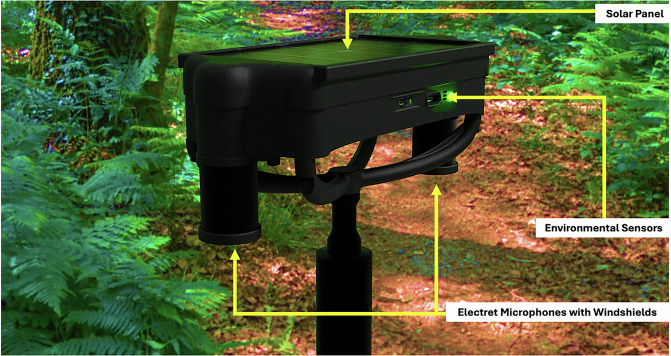
BuzzCam with sensor locations shown. The system can be mounted to a standard tripod so the height is adjustable.

**Fig. 2:**
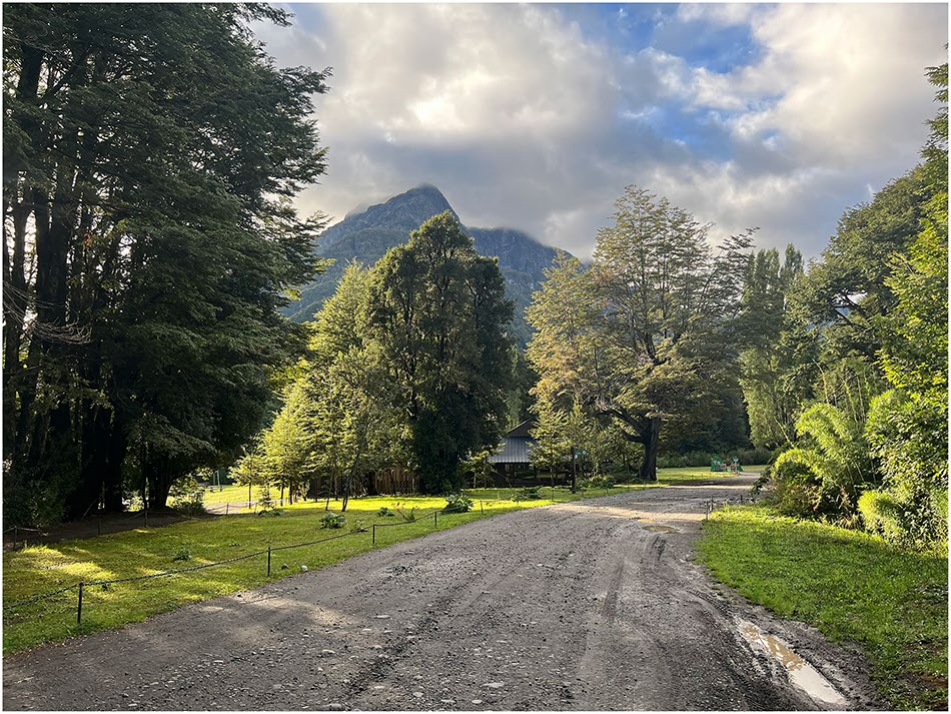
Road nearby sensor locations 1 through 3 (reference Fig. 4).

**Fig. 3:**
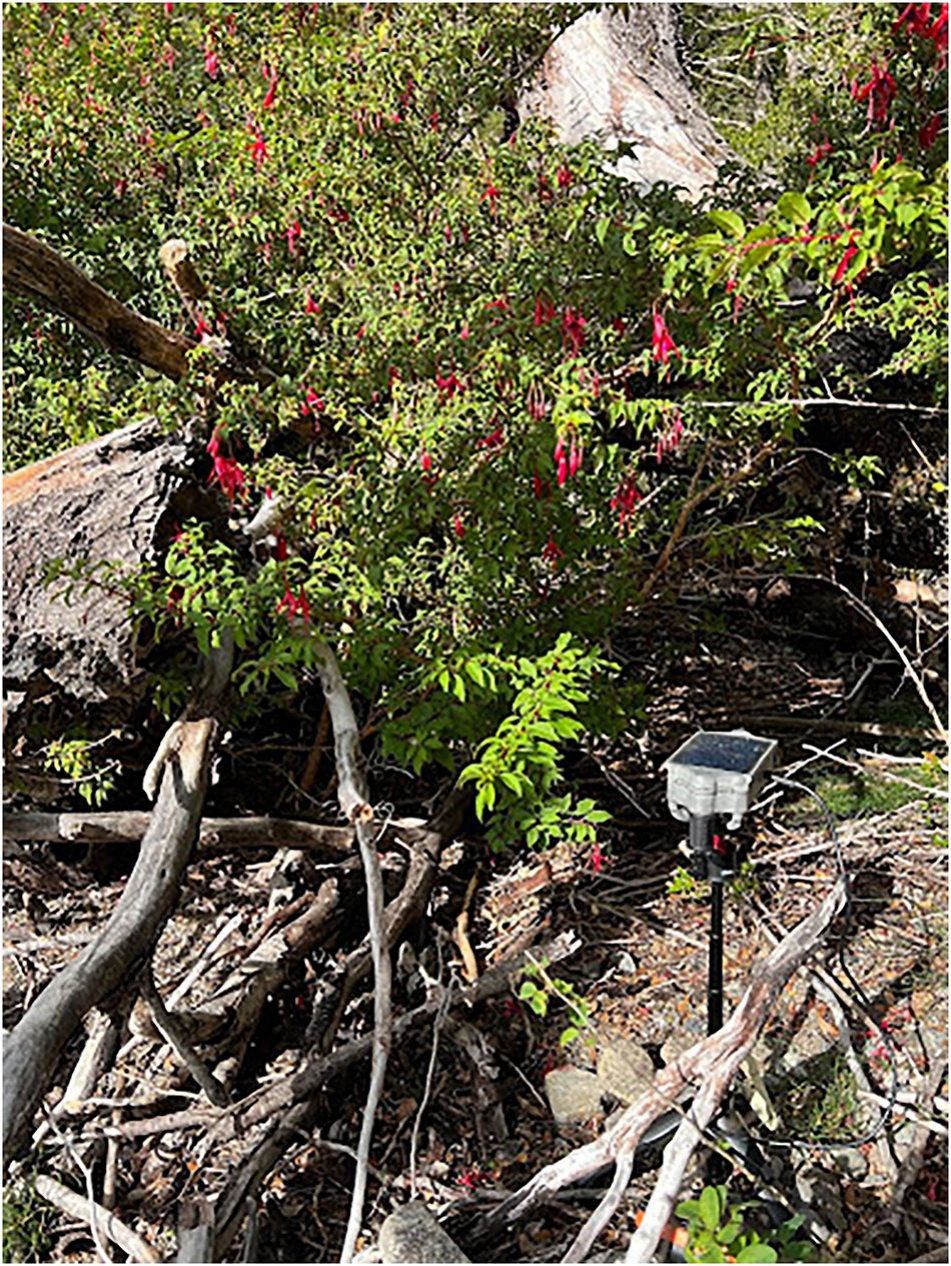
Example of sensor deployment. BuzzCam sensor pictured near Fuchsia bush.

**Fig. 4:**
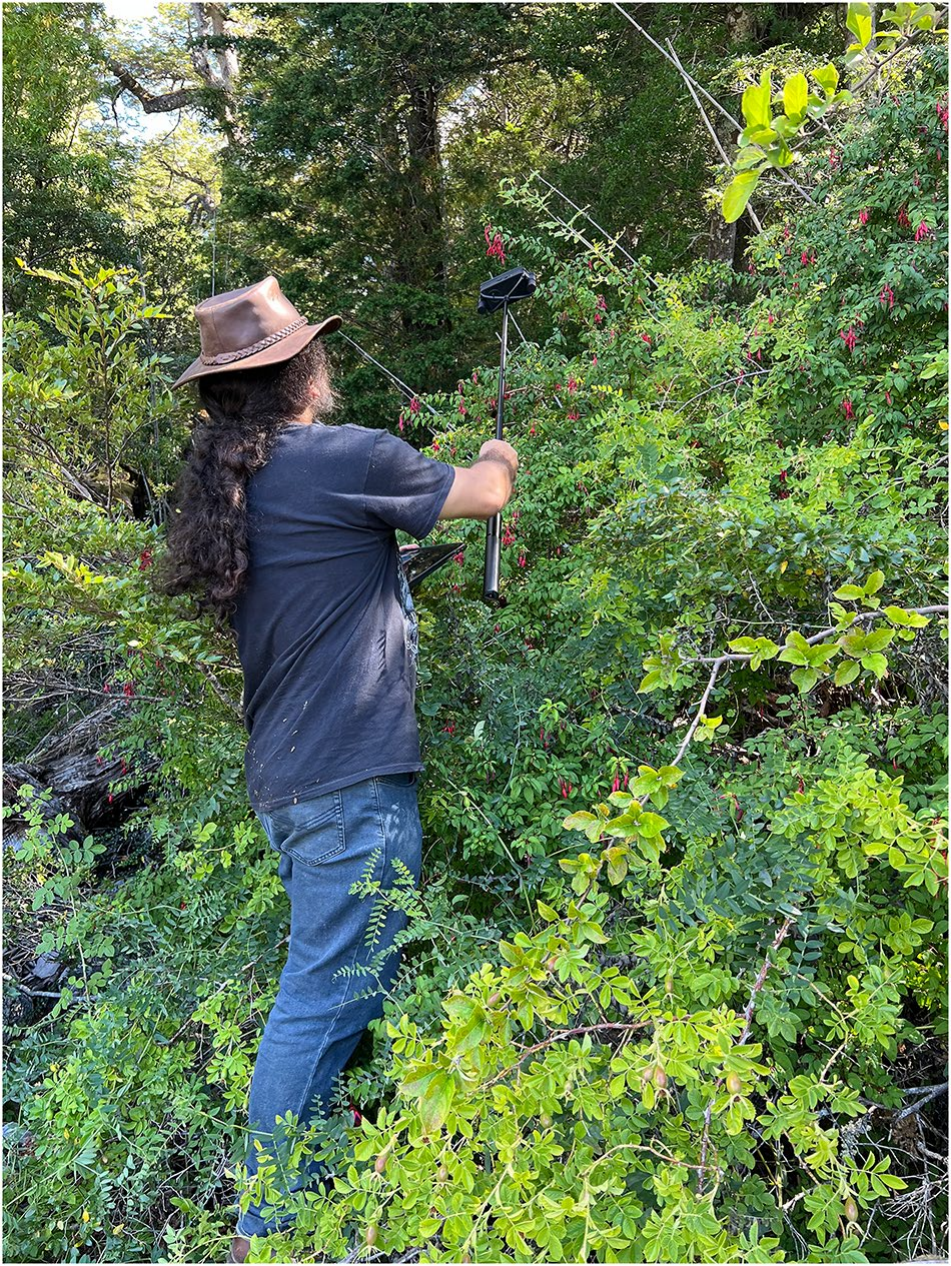
Example of dynamically-positioned microphone.

**Fig. 5:**
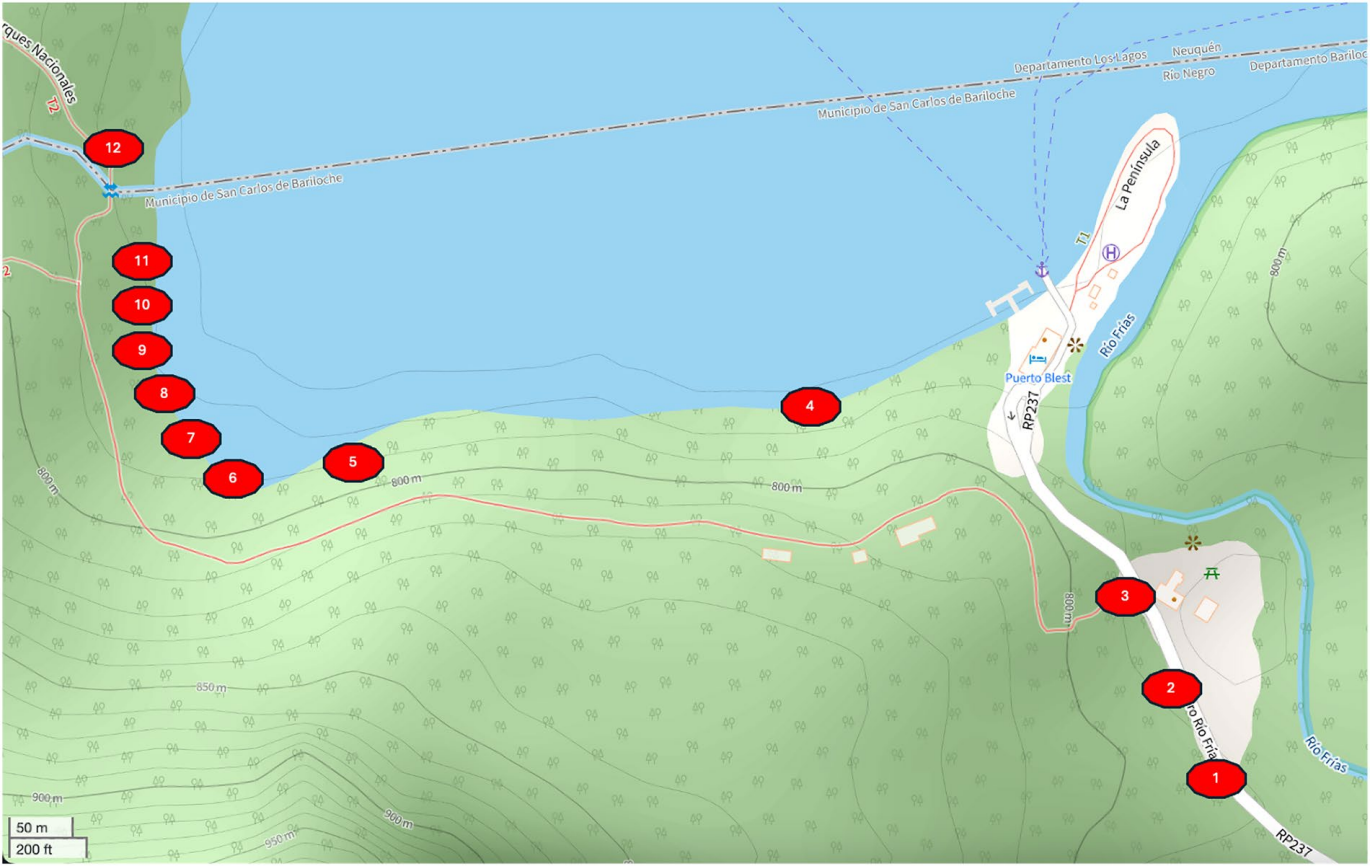
Locations of test sites in the Puerto Blest Area. Numbers represent numbered field sites.

We built a total of 9 stereo recorders, called BuzzCam, each identified by a unique 4-character hexadecimal identifier (Table 2, Fig. 1). Over the course of the six data collection days, the team first identified several locations where native and invasive bees were observed, then placed the device at those locations Table 3, all at the same height (Table 4, Fig. 3). Some locations were designated more than one recorder due to their larger size. Given the season and ecology of Puerto Blest, each location was selected to include a blooming Fuchsia shrub to attract bumblebees. Locations 1 through 3 were off a road and more inland (Fig. 2, Table 4). The nearby vegetation includes a mix of tall trees, shrubs, and grassy areas. Locations 4 through 12 were located within an ecotone and were topographically similar, with locations 11 and 12 uniquely characterized by distinct sound profiles due to their proximity to a stream. An example of a location on the bank of Nahuel Huapi Lake is shown in Fig. 3. For each location, GPS coordinates were taken using an iPhone 14 Pro with an accuracy of 5 meters in degrees, minutes, and seconds format (DMS). The earlier locations (i.e., 1-3) were near tourist and motor vehicle or generator activity, which polluted audio data Fig. 4. In response, we decided to focus on several Fuchsia shrubs on the shoreline of Nahuel Huapi Lake for the later locations (Fig. 5). These shrubs were found to have the greatest density of *B. dahlbomii* activity, after witnessing minimal activity near inland shrubs, despite some activity while scouting field sites. All recordings are during day-time hours except for tests 2.2.1 and 3.2.1 which were left overnight. It is also important to note that only worker and male *Bombus* activity was observed, with no queens present due to it being late in the season. We also did not observe any *Bombus ruderatus* in the area.

**Table 2:** Sensor IDs for each BuzzCam system.

BuzzCam Sensor IDs
50DD
55DA
55E0
546C
548C
55E8
5AB2
5ACD
49C2

**Table 3:** Results from Acoustic Data Validation.

		Samples	
		1-second	10-second
**Negative**	**Terrestris**	4,812	278
	**Dahlbomii**	6,907	404
**Positive**	**Terrestris**	5,455	1,023
	**Dahlbomii**	4,572	1,020

**Table 4:** Sensor deployments and locations of test sites in the Puerto Blest Area.

Locations	Tests	Static Sensors	GPS Coordinates	Notes
1	1.1	546C, 548C, 55E0, 50DD, 55DA	41°1′39″ S 71°48′45″W	
	1.2	50DD, 55E0, 55DA		
2	1.1	55E8	41°1′38″ S 71°48′45″W	
	1.2	55E8		
3	1.1	49C2, 5ACD	41°1′34″ S 71°48′47″W	
4	2.1	5ACD	41°01′31″S 71°48′58″W	
5	2.1	5AB2	41°1′32″S 71°49′15″W	
	2.2	50DD		Overnight recording during rainstorm
6	1.3	548C	41°1′31″S 71°49′20″W	
	2.1	50DD		
7	1.3	49C2	41°1′30″S 71°49′20″W	
	2.1	55E8		
	3.1	50DD		
	4.1	55DA		
	5.1	49C2		
	6.1	55E8		
8	1.3	5ACD, 548C	41°1′29″S 71°49′20″W	
	2.1	55E0		
	3.1	55DA		
	3.2	55E8		Overnight recording
	4.1	5AB2		
	5.1	5ACD, 548C		
	6.1	55DA, 55E0		
9	1.3	546C	41°1′29″S 71°49′20″W	
	2.1	55DA		
	3.1	55E0		
	4.1	55E8		
	5.1	50DD, 55E0		
	6.1	49C2, 546C		
10	1.3	546C	41°1′28″S 71°49′20″W	
	2.1	548C		
	3.1	55E8		
	4.1	5ACD		
	5.1	55DA		
	6.1	5ACD		
11	1.3	546C	41°1′27″S 71°49′20″W	
	2.1	546C		
	3.1	5AB2		
	4.1	548C		
	5.1	5AB2		
	6.1	5AB2		
12	2.1	50DD	41°1′25″S 71°49′20″W	Nearby waterfall

(Tests are defined as day session).

During the initial deployments, *B. dahlbomii* activity was observed to be relatively low with only one observation every several minutes. In response, we adopted a hybrid approach where we annotated both microphone systems that were stationary (static) (Table 4), as well as attached microphones to a mobile tripod that we held closer to any observed *B. dahlbomii* activity (dynamic) (Fig. 4). In our dataset, for any given annotation, we label if that annotation was done on a static or dynamic microphone. For the dynamic method, more footsteps are heard in the audio as annotators traverse the rocky terrain to better position the microphone. The test sessions that included dynamically positioned microphones are 3.1, 4.1, 5.1, and 6.1 and a separate folder labeled “dynamic” exists in the repository for those respective sessions.

The team recorded roughly 250 hours of uncompressed (.WAV) stereo data in which a subset of that data was annotated in real-time by human observers. The dataset includes recordings over various weather conditions and hours, including a few overnight recordings where we tested the longevity and robustness of the recording system.

Prior to publishing the uncompressed raw acoustic data, we reviewed all the recorded audio and removed any intelligible human speech, as the recorders captured conversations from passersbys. The team manually scanned the spectrograms for each recorded audio file, selected segments where the power within the spectrogram correlated with human speech frequencies, listened to confirm it was human speech, and silenced the segment if human speech was detected. For privacy concerns, we silenced those sections of audio using Audacity. By silencing, rather than deleting sections of audio, we preserved the true length of the recordings for reference back to world time.

## Data Records

This dataset is available on Figshare, including raw acoustic and environmental data, processed excerpts, and Amazon Mechanical Turk (ATurk) results^43^. ATurk is an online crowdsourcing platform that enables researchers to enlist a diverse pool of human workers to perform tasks that require human intelligence, such as data labeling or annotation. The team used ATurk to further validate if field-annotated excerpts all had noticeable bee buzzes. The processed excerpts are the segments of audio that have been validated to have bee buzzes and further annotated for corresponding species. Five sections of data records exist within this repository and their descriptions are as follows.

### Collected Data

The field recordings are labeled using a two digit label scheme where the first digit corresponds to the day, 1 (March 10th, 2024) to 6 (March 15th, 2024). The second digit maps to the session in that day that the recording was taken place. On several days, we conducted multiple recording sessions either because of the weather or to reposition the microphones away from increased human activity (e.g., tourists). For example, a label of 2.2 means the recording was taken on the second day (March 11th, 2024), in the second session.

**Fig. 6:**
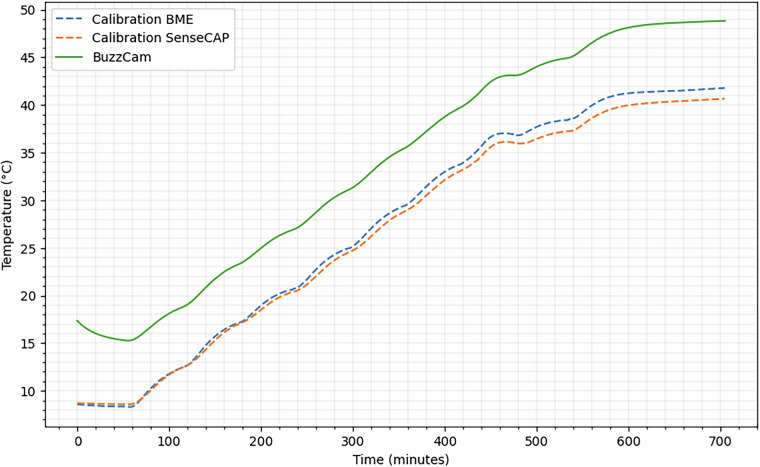
Temperature calibration among BuzzCam, the sensor device used for field recordings, an external BME688 sensor identical to the one within BuzzCam, and a Seeed Studio SenseCAP, all placed at the same elevation within a controlled climate chamber.

**Fig. 7:**
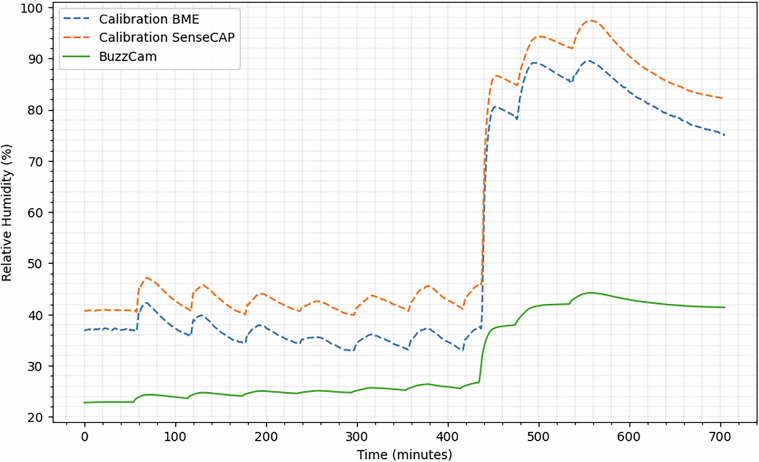
Humidity calibration among BuzzCam, the sensor device used for field recordings, an external BME688 sensor identical to the one within BuzzCam, and a Seeed Studio SenseCAP, all placed at the same elevation within a controlled climate chamber.

**Fig. 8:**
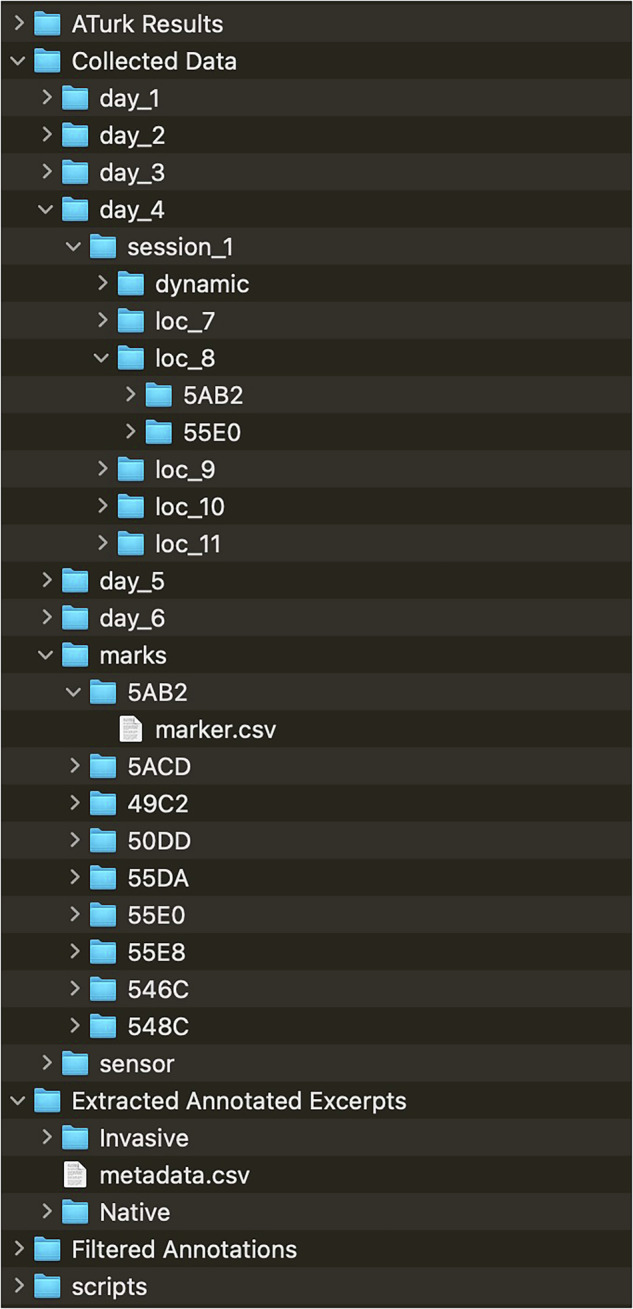
Example file structure of repository.

The Collected Data section has all the raw data from the fieldwork collection with supporting metadata about file length and timestamps explained in collected_data_summary.xlsx. The structure of the folders for the acoustic data follows the test designations from Table 4. For example, test 4.1 in location 8 for sensor 5AB2 is located in the day_4/session_1/loc_8/5AB2 directory (Fig. 8). Each session has one or more .wav files within it that are named audio_{index}_edit.wav where the index starts from zero and continues until the recording session is complete. Each incremental index is a continuous of the one prior and were split up during recording so that no file is larger than 2 GB. The approximate start time for each recording is within the same folder as the .wav file for any given session, logged under the orientation.csv file. That file includes the start time of the recording, as well as the orientation of the device. The orientation is not relevant for these recordings, as we had to slightly manually reposition microphones at times due to an unstable footing.

Within the collected data folder is a marks folder which includes subdirectories for each recorder device, named by the individual device IDs. Within a device folder exists a marker.csv file that for every row, has the following:

- *timestamp*: Unix epoch synchronized to UTC time
- *ms_from_start*: milliseconds since the system has been turned on
- *beep_enabled*: if the system’s piezoelectric beeper was turned on for this annotation. If “1”, the beep will be heard in the acoustic data as a loud chirp (4.4-5.6kHz, 300ms)
- *annotation*: a written text from the user annotating on the iOS device. “Native” indicates observed *Bombus dahlbomii* activity while “Invasive” indicates observed *Bombus terrestris* activity.

Lastly, within the collected data section, the sensor folder includes subdirectories for each device ID where a CSV file records all the raw environmental sensor readings reported from the BME688 sensor over the duration of the data collection. Each row of the sensor.csv file has the following:

- *timestamp*: Unix epoch synchornized to UTC time
- *time_sensor*: a timestamp reported by the sensor itself after each reading
- *time_ms_start*: milliseconds since the system has been turned on
- *signal*: sensor reading for the given sensor id
- *signal_dim*: a value reported by the BME688 which is redundant and can be neglected for this experiment
- *sensor_id*: sensor ID maps to a particular sensor (e.g., temperature, humidity, equivalent CO_2_) (see BME688 datasheet)
- *accuracy*: the accuracy reported by the BME688 (see BME688 datasheet)

### Extracted Annotated Excerpts

Using the annotations from the marks folder, we created a script (annotation_parser.ipynb). This script removed 10-second stereo snippets of audio that were labelled “Native” or “Invasive”, centered on the annotation time. For this dataset, we use “Native” and “Invasive” as labels to describe *Bombus dahlbomii* and *Bombus terrestris*, respectively. Within the filtered folder, a metadata.csv file contains the following information:

- *type*: “Native” or “Invasive” annotation
- *file_name*: name of 10-second audio excerpt
- *origin*: which raw audio file the excerpt is from
- *seconds*: number of seconds within the original raw audio file that the excerpt was extracted from
- *mic_type*: “static” for a statically positioned microphone or “dynamic” for one that was actively moved to locations with observed bees

### ATurk Results

The ATurk results are located here in CSV format with one column specifying the filenames of the extracted 10-second annotated excerpts. The second column specifies a number out of 3 where 0 means no participants voted that the audio file had a buzz while 3 means all participants voted there was a perceived buzz.

### Filtered Annotations

In this section, we present the results of the Technical Validation, where we used a series of automated and manual methods to remove excerpts that were mislabeled or contained silenced sections of audio from when we removed intelligible human speech from the raw recordings. Using the field annotations, we filtered the Extracted Annotated Excerpts into two new sections: Negative_10sec and Positive_10sec. In the Positive_10sec folder, we have data with a high certainty of containing a buzz, while in the Negative_10sec folder, there are no perceived buzzes. We also discretized our original excerpts into finer 1-second chunks and repeated the process of validating the contents, categorizing them into their respective folders: Positive_1sec and Negative_1sec. All files within this section have a prefix of “Native” or “Invasive”, indicating the original in-field annotation. In-depth details of this process are described in the Technical Validation section.

### Weather Station

In this folder is a text file called Puerto_Blest_Weather_Station.txt which is the raw data provided by the local weather station in Puerto Blest. The file has data at an hourly resolution references to local time (UTC-3) from January 1st, 2024, to May 21, 2024. The data includes temperature, humidity, wind speed, wind direction, pressure, and rain rate.

## Technical Validation

### Acoustic Data

We extracted all the annotations with their respective timestamps, along with timestamped audio and environmental recordings. These annotations were used to extract 10-second sound clips centered around when the annotation was triggered, resulting in 6,162 10-second recordings that were separated by native and invasive labels. After randomly sampling 150 recordings, we observed that some of the annotated recordings did not include buzzes. We attribute this to errors in the annotation process, such as a button on the application being clicked when a bee was observed near the recorder but not audibly buzzing, or the buzz occurring too far from the recorder. Increased distance from the recorder raises the probability that the buzz was lost in the noise from Nahuel Huapi Lake, human activity, wind-related activity (e.g., branches and leaves moving), and other wildlife sounds.

Given that some of the annotated acoustic excerpts didn’t have the expected buzzes, we further annotated the excerpts using (ATurk). The team applied for MIT Institutional Review Board (IRB) approval because the further annotations required human subjects, and the study was exempt (E-5859). IRB approval is necessary to ensure the ethical treatment of participants and to comply with regulations when involving human subjects in research. Prior to posting on ATurk, we converted the uncompressed stereo audio files to compressed MP3 format to reduce the file size and mitigate any issues annotators might have with internet bandwidth constraints. We also removed any excerpts that had silenced portions of audio data during our initial preprocessing. This resulted in 5,880 acoustic data files, which were randomly divided into multiple groups of 150 samples and submitted to ATurk to be annotated as “buzz” or “no buzz” by three human annotators per group. This resulted in the following data descriptor: buzz_count: [0,3], where 0 means no participants voted that the audio file had a buzz, and 3 means all participants voted that a perceived buzz exists within a given 10-second audio excerpt. Using ATurk increased the robustness of our dataset but introduced a new source of error. After randomly sampling the ATurk results, we found that annotators occasionally mistook natural sounds, such as subtle waves crashing on the shore, for bee buzzes. This was not a source of error for our human observers in the field which had visual cues. To address this, we employed an additional filtering approach using an Audio Spectrogram Transformer Model (AST)^44^ trained on the Audio Set^45^ dataset with labels related to bees, buzzes, and similar insect sounds. Prior to using the AST, we further divided our 10-second excerpt data into 1-second sound snippets with a 0.5-second overlap. We then removed any silenced snippets. This process generated 19 one-second audio snippets from each 10-second file, resulting in 110,907 1-second uncompressed stereo audio snippets.

The Audio Set labeled data that AST is trained on includes 527 labels, such as insects, animals, and environmental sounds, but only labels related to bee buzzes were targeted for validation of this dataset. For the positive results of the annotated datasets, we sought to eliminate any clips that have either mislabeled data (i.e., no buzzes) or ambiguous sounds that can’t be reliably perceived as buzzes. The logic we used for positive filtering of the 1-second samples is as follows: First, we selected only the data where at least 1 ATurk annotator believed there to be a bee buzz. Then, of those clips, we only selected those where a specific label ‘Bee, wasp, etc.’ is included in the top 5 categories output from the AST model for each clip. For the selected 10-second positive clips, we only chose those that had 2 or more of their 19 1-second snippets classified as positive 1-second buzz samples.

We knew that a subset of the annotated dataset was misannotated from random sampling, likely due to annotator fatigue in the field. However, using a similar method to our positive dataset validation, we created a negative training set for model creation. From the annotated data, we selected clips where at least 2 ATurk annotators perceived no bee buzzes. We then processed these clips through AST and selected the ones where none of the top 5 AST output categories related to bees. Through our testing, these bee-related categories are: ‘Bee, wasp, etc.’, ‘Fly, housefly’, ‘Insect’, ‘Buzz’, ‘Fart’, ‘Snoring’, ‘Snort’, ‘Zipper (clothing)’, and ‘Mosquito’. For the 10-second negative excerpts, we only selected those where all the 1-second snippets originating from a 10-second excerpt were part of the negative set.

This process resulted in 10,027 positive and 11,719 negative 1-second uncompressed stereo audio snippets. The complete breakdown of the filtered annotation results is shown in Table 3, including the balance between *Bombus dahlbomii* and *Bombus terrestris*.

### Environmental Sensor Data

The environmental sensors within the BuzzCam devices are placed behind gas-permeable fabric, which could influence the sensor readings. Environmental changes will still be observed, but the sensed magnitude and derivatives may differ from actual ambient readings. To compensate for this, we calibrated our BuzzCam temperature sensors in a climate chamber where we regulated the temperature from 0 to 45°C in 5°C increments. As a reference, we placed a BME688 sensor, the same used within BuzzCam, alongside BuzzCam without any gas-permeable fabric covering it. We also used a separate Seeed Studio SenseCAP as a second reference. As shown in Fig. 6, there was a significant difference between the BuzzCam and reference sensors; however, this can be compensated for by applying an offset to the BuzzCam. The difference between the BuzzCam and the external BME688 was 6.4°C  ±0.5, while the difference between the BuzzCam and the SenseCAP was 7.1°C  ±0.7.

As shown in Fig. 7, the humidity differences between the BuzzCam and the reference sensors in a controlled chamber were more substantial. The variability observed between the two calibration sensors is within their  ± 5% error specification. Although the BuzzCam system can detect humidity changes in the environment, its readings are approximately 57.9% of those from the reference BME sensor and 51.8% of the SenseCAP. Given that the sensor is sensitive to humidity changes, the collected data can be used in multi-modal modeling that leverages environmental dynamics to enhance detection accuracy. However, future iterations of BuzzCam require increased cross ventilation across the sensors to improve sensing accuracy.

## Usage Notes

The dataset is structured to easily integrate into researchers’ modeling work. For instance, the Filtered Annotation section can be used to train a model sensitive to bee buzzes or explore the feasibility of creating a model capable of distinguishing species using acoustic data. Environmental data from the Collected Data section enables the creation of a multi-modal model that considers environmental parameters for increased robustness. The individual sensor environmental data can also be combined with the provided weather station data to account for broader environmental characteristics not available at the individual sensor level (e.g., wind speed). Note that the weather station was located farther inland from Nahuel Huapi Lake, so the environmental readings may differ from the locally sampled readings along the shoreline.

Please be cautious when developing a buzz acoustic detector using this dataset as it primarily includes buzzes from *Bombus dahlbomii* and *Bombus terrestris*. This limitation suggests that models trained solely on this data might have restricted generalizability to other bee species. In addition, if using this dataset to quantify unique bee buzzes, users should take into consideration recordings that are made by multiple units within the same location at the same time. Multiple recorders could potentially record the same bee buzz, although the acoustic signal unique to each device would give spatial differences.

If a researcher wishes to reference the filtered excerpts to the original raw data, the metadata.csv file within the Extracted Annotated Excerpts section can be used to relate the excerpts to a location in the raw data. This facilitates training of machine learning networks with attention mechanisms that can utilize the raw continuous acoustic data as input.

The weather station data can be used as an additional data point for the local environment and as an input into a multi-modal model that incorporates environmental parameters, such as wind speed, into a probabilistic model of nearby buzz occurrences.

## Data Availability

All the Python scripts used to convert the raw data into the shorter, annotated excerpts are included in the repository, under the scripts folder^43^. This code includes Jupyter notebooks with Python analysis workflows that can be easily reproduced. We also include the requirements.txt file to recreate the Python environment that was used for the processing of the data.
